# Mucinous ovarian carcinoma in four teenage girls with fatal outcomes

**DOI:** 10.3389/fonc.2026.1839124

**Published:** 2026-06-03

**Authors:** Lisa Feinstein

**Affiliations:** Camelot Animal Hospital, Davie, FL, United States

**Keywords:** mucinous ovarian adenocarcinoma, mucinous ovarian cancer, mucinous ovarian cystadenoma, ovarian cysts, ovarian mucinous adenocarcinoma, pediatric ovarian masses, pediatric ovarian neoplasm

Mucinous ovarian carcinoma (MOC) is a rare gynecological cancer that occurs in younger women. There are rare case reports in teenagers. In “Tp53 and KRAS co-mutations are associated with worse outcomes in mucinous ovarian carcinomas,” Zhang et al. (2025) showed a correlation between TP53 and KRAS co-mutations and worse outcome and younger age in women with MOC even those diagnosed at stage 1. This case series of 4 young women concurs with these findings and highlights the need for more targeted treatment for MOC.

Four female patients were collected from the Mucinous Ovarian Cancer group on Facebook. The young women were diagnosed with primary ovarian Mucinous Ovarian Carcinoma (MOC) when they were teenagers (14 to 19 years old) and suffered a fatal outcome in the last four years (2022-2026). Their mothers provided the data from their medical reports. 

## Introduction

All four girls were teenagers when diagnosed (14 to 19 years old) (**Table 1**). They presented with abdominal pain, and imaging identified large ovarian cysts. The ovarian cysts ranged from 14 to 23 cm. Three of the four girls were operated on initially by a gynecologist since the gynecologist did not suspect mucinous ovarian carcinoma. The youngest girl (patient 3) who was 14 years old, was operated on by a pediatric surgeon. The Helsinki pediatric oncology team did initially suspect cancer since there was ascites on pre-surgical imaging of the 13 x 14 x 10 cm cyst, but they did not suspect mucinous cancer.

**Table 1:** Patient characteristics, staging, and initial treatment (2022–2026).

Patient	Country/state	Age (years) at diagnosis and year of diagnosis	Initial surgery	Initial surgeon	Stage	Chemotherapy type	Age at death (years) and year of death
1	Florida, USA	18 (2023)	Cystectomy	Gynecologist	Stage 3a1 (Omentum Abdominal fluid)	Folfox	19 (2024)
2	Texas, USA	18 (2022)	Salpingo- oophorectomy	Gynecologist	Stage 2a (Uterus)	HIPEC (Cisplatin) cytoreduction, Folfox	20 (2024)
3	Finland	14 (2024)	Salpingo- Oophorectomy	Pediatric surgeon	Stage 1c (Spillage)	Carbo/Taxol	15 (2026)
4	Illinois, USA	19 (2022)	Cystectomy	Gynecologist	Stage 3a2 (Omentum abdominal fluid)	Carbo/Taxol	20 (2023)

Two of the young women had cystectomies at their initial surgery, where MOC was identified on pathology after surgery, and two of the young women underwent salpingo-oophorectomy at initial surgery (**Table 1**). The three young women who were operated on by a gynecologist then underwent follow-up staging surgery approximately 6 to 8 weeks later with a gynecological oncologist. Two were staged as stage 3a, as MOC was found in the peritoneal fluid and in the omentum. One was staged as stage 2a, as MOC was found in the cul-de-sac and uterus. Patient 3 (the 14-year-old) was staged at her initial surgery, as cancer was suspected. She was stage 1c due to spillage during the surgery.

The pathology of the four women’s tumors was expansile (**Table 2**). Patient 1 and 4 had tumors described as expansile, well-differentiated with intraepithelial carcinoma. Patient 2’s tumor was similar but showed moderate differentiation. The MOC tumor biomarkers showed TP53 and KRAS co-mutations in all patients with known tumor genetics. Patients 2 and 3 had TP53 and KRAS G12D mutations, and patient 1 had TP53 and KRAS G12V mutations. CDKN2A mutations were also identified in patient 1 and patient 3, and HER2 mutation was expressed in patient 1 and patient 2. The family of patient 4 did not have tumor genetic data available to verify mutations.

**Table 2:** MOC tumor characteristics.

Patient	Size of cyst (cm)	Prior cyst or reproductive disease	Initial suspicion of MOC before surgery	Pathology	MOC tumor biomarkers
1	18	Mucinous adenoma ipsilateral ovary cystectomy 18 months prior	No	Expansile well differentiated with intraepithelial carcinoma	TP53, KRAS G12V, CDKN2A, HER2 (intermediate)
2	23	No	No	Expansile, Moderately differentiated with Intraepithelial carcinoma	TP53, KRAS G12D, HER2
3	14	No	No (Malignancy was suspected, but doctors did not suspect MOC)	Expansile	TP53, KRAS G12D, CDKN2A
4	23	Endometriosis	No	Expansile well differentiated with intraepithelial carcinoma	Unknown

All four patients were treated with chemotherapy after their fertility-sparing (unilateral oophorectomy) staging surgery (**Table 1**). Patient 2 underwent HIPEC cytoreduction with cisplatin (200mg/m2) followed by Folfox (folinic acid, fluorouracil, and oxaliplatin) chemotherapy for six cycles. Patient 1 had Folfox chemotherapy for six cycles after staging. Patient 4 had Carbo/Taxol (Carboplatin+Paclitaxel) for six cycles. Patient 3 had four cycles of Carbo/Taxol, which was changed to Docetaxel (Docetaxel-Carboplatin) for the final two cycles due to a severe allergic reaction.

All four patients experienced recurrence, ranging from 5 months to 20 months after diagnosis (**Table 3**). Patient 1 had confirmed recurrence with omental thickening on CT and a rise in CA125 to 54 at 6 months after staging surgery, within 2 weeks of completing Folfox chemotherapy. Patient 4 had confirmed recurrence at 5 months, shortly after finishing six cycles of Carbo/Taxol, when CT showed a large cyst on the remaining ovary and a liver nodule. Patient 3 had an elevated CA125 (39) at 8 months, with recurrence confirmed at 9 months after staging surgery, when MRI showed abdominal wall metastasis and tumor nodules in the pelvis. Patient 2 had abdominal pain at 18 months, which was initially thought to be adhesions on CT imaging. Recurrence was not confirmed until 20 months, when CT imaging showed abdominal nodules, which were confirmed to be metastatic, poorly differentiated MOC on biopsy.

**Table 3:** Recurrence and death.

Patient	Time from diagnosis to recurrence (months)	Organs where recurred	Recurrence treatment	Time from diagnosis to death (months)
1	6	Omentum, appendix, ascites, peritoneum, and intestinal tract	HIPEC (Cisplatin) cytoreduction, Enhertu, gastrostomy tube	14
2	20	Peritoneum, liver, intestines, and ascites	RMC-6236 (Daraxonrasib), Biliary stent	30
3	8	Peritoneum and on the other ovary	Nab-Paclitaxel, Bevacizumab withTraverinib, then Nivolumab- Etinostat	16
4	5	Liver, kidney, other ovary, and ascites	Folfox, Oophorectomy, Ileostomy, Colostomy	13

Chemotherapy was administered for recurrence in all four patients (**Table 3**). Patient 1 underwent HIPEC cytoreduction with cisplatin (200 mg/m2). MOC was noted all over the abdomen at surgery (peritoneal carcinoma index of 39 and MOC on the entire gastrointestinal tract). She developed bowel obstruction within 3 weeks after surgery. Since patient 1’s tumor showed HER2 mutation, Enhertu was administered for three cycles, but it failed to reduce the tumor burden. After recurrence, patient 2 received Daraxonrasib, a KRAS targeted drug, for four months; however, the MOC tumor continued to grow and created ascites and blocked gallbladder flow, which required a stent placement. Patient 3 received weekly nab-paclitaxel with monthly bevacizumab infusion for three months, with traverinib added later for one month. Since the MOC tumor continued to grow, patient 3 was enrolled in an immunotherapy trial where she received weekly infusions of nivolumab-etinostat. After recurrence, patient 4 underwent salpingo-oophorectomy for a tumor on her other ovary 7 months after staging and was treated with Folfox chemotherapy. She developed bowel obstruction and required ileostomy and colostomy.

All four women died of disease 13 to 30 months after diagnosis (**Table 3**). All experienced tumor growth in the abdomen that created ascites, intestinal blockage symptoms, and an inability to eat and drink. MOC also spread to the chest cavity in patients 1 and 2, resulting in malignant pleural effusions. All deaths were attributable to progressive MOC.

## Discussion

The four young women included in this case series were diagnosed with MOC as teenagers and died of disease within approximately 2 years of diagnosis. Their initial gynecologists or surgeons did not suspect their large cyst to be MOC before the initial surgery, highlighting the need for increased awareness within the gynecologic community that teenagers can harbor MOC in large ovarian cysts. The four young women underwent fertility-sparing, salpingo-oophorectomy staging surgery, and standard chemotherapy for six cycles. Despite the pathology showing an expansile growth pattern, MOC recurred and acted very aggressively. The MOC tumor from three girls with known tumor genetics showed TP and KRAS co-mutations. Zhang et al. (2025) demonstrated that TP53 and KRAS co-mutations are associated with worse outcomes in mucinous ovarian carcinomas. Women with tumors that carried the mutations were more likely to have recurrence and worse prognosis, even if they were younger and diagnosed at stage 1. This small case series supports the findings in Zhang et al.’s study. These observations underscore the need for targeted treatments, especially in young women who are undergoing fertility-sparing surgery. They also demonstrate that tumor biomarkers are important in guiding treatment strategy, as an expansile (versus infiltrative) pattern does not guarantee benign behavior.

